# Psb34 protein modulates binding of high-light-inducible proteins to CP47-containing photosystem II assembly intermediates in the cyanobacterium *Synechocystis* sp. PCC 6803

**DOI:** 10.1007/s11120-022-00908-9

**Published:** 2022-03-13

**Authors:** Parisa Rahimzadeh-Karvansara, Guillem Pascual-Aznar, Martina Bečková, Josef Komenda

**Affiliations:** Laboratory of Photosynthesis, Centre Algatech, Institute of Microbiology of the Czech Academy of Sciences, Opatovický mlýn, 37981 Třeboň, Czech Republic

**Keywords:** Photosystem II, Photosynthesis, CP47, High-light-inducible protein

## Abstract

Assembly of photosystem II (PSII), a water-splitting catalyst in chloroplasts and cyanobacteria, requires numerous auxiliary proteins which promote individual steps of this sequential process and transiently associate with one or more assembly intermediate complexes. In this study, we focussed on the role of a PSII-associated protein encoded by the *ssl1498* gene in the cyanobacterium *Synechocystis* sp. PCC 6803. The N-terminal domain of this protein, which is here called Psb34, is very similar to the N-terminus of HliA/B proteins belonging to a family of high-light-inducible proteins (Hlips). Psb34 was identified in both dimeric and monomeric PSII, as well as in a PSII monomer lacking CP43 and containing Psb28. When FLAG-tagged, the protein is co-purified with these three complexes and with the PSII auxiliary proteins Psb27 and Psb28. However, the preparation also contained the oxygen-evolving enhancers PsbO and PsbV and lacked HliA/B proteins even when isolated from high-light-treated cells. The data suggest that Psb34 competes with HliA/B for the same binding site and that it is one of the components involved in the final conversion of late PSII assembly intermediates into functional PSII complexes, possibly keeping them free of Hlips. Unlike HliA/B, Psb34 does bind to the CP47 assembly module before its incorporation into PSII. Analysis of strains lacking Psb34 indicates that Psb34 mediates the optimal equilibrium of HliA/B binding among individual PSII assembly intermediates containing CP47, allowing Hlip-mediated photoprotection at all stages of PSII assembly.

**Supplementary Information:**

The online version contains supplementary material available at 10.1007/s11120-022-00908-9.

## Introduction

Oxygenic photosynthesis is a complex natural process during which the energy from sun radiation is deposited into energy-rich compounds and reduced biosynthetic cofactors, which are utilized for the conversion of carbon dioxide into saccharides and other organic molecules. The crucial stage of photosynthetic energy conversion is light-driven photosynthetic electron flow, which is fed by electrons from Photosystem II (PSII), a large pigment-binding protein complex of cyanobacteria, algae, and higher plants. This complex uses water as an electron donor and releases molecular oxygen as a by-product (Barber 2003). PSII is very complex, containing nearly two dozen protein subunits, a large number of pigments and other organic and inorganic cofactors (Umena et al. 2011). The biogenesis of PSII is thought to involve a stepwise interconnection of several pigment–protein intermediate complexes, here called modules, each containing one of the large pigment-binding proteins (D1, D2, CP47 and CP43), adjacent small subunits, pigments, and other cofactors, as well as auxiliary proteins (Komenda et al. 2012b). In the cyanobacterium *Synechocystis* sp. PCC 6803 (hereafter *Synechocystis*), assembly of PSII has been shown to begin with the association of the D1 and D2 modules (D1_mod_ and D2_mod_) consisting of D1-PsbI and D2-cytochrome b_559_ pairs, respectively. The resulting PSII reaction centre complex (RCII) binds the CP47 module (CP47_mod_) to form the PSII core complex lacking CP43 (RC47; Komenda et al. 2004; Boehm et al. 2012). Subsequently, addition of the CP43 module (CP43_mod_) results in the formation of a monomeric PSII core complex (PSII(1); Komenda et al. 2012a). PSII(1) then undergoes the process of light-driven formation of the oxygen-evolving complex (OEC) containing the CaMn_4_O_5_ cluster (Bao and Burnap 2016) and the oxygen-evolving enhancer (OEE) subunits PsbO, PsbU and PsbV, which shield the cluster (Roose et al. 2016). The final step of PSII assembly appears to be dimerization of PSII(1) into the PSII dimer (PSII(2); Komenda et al. 2012b).

PSII assembly in cyanobacteria is assisted by a large number of auxiliary protein factors playing more or less important roles during various stages of the process. Some of the early-stage factors like Ycf48 (Yu et al. 2018) and RubA (Kiss et al. 2019) are important for insertion of chlorophyll (Chl) into RCII. Others, like Psb28 (Dobáková et al. 2009) or Psb27 (Nowaczyk et al. 2006; Roose and Pakrasi (2008); Komenda et al. 2012a), participate in the later stage when the PSII core complex is formed and is to be converted into the oxygen-evolving dimer. So-called high-light-inducible-proteins (Hlips) belong to auxiliary factors involved in both early and late stages of the assembly process (for review See Komenda and Sobotka, 2016). Genes encoding these single transmembrane helix proteins form a family that is present in most cyanobacterial species and are expressed under high irradiance and other stress conditions (Dolganov et al.^1995^). The amino acid sequence of their transmembrane helix is quite similar to that of the first or third helix of plant light-harvesting complexes (Montané and Kloppstech 2000), including the domain binding Chl (Cab domain). Therefore, Hlips are also alternatively called small cab-like binding proteins (Scps, Funk and Vermaas 1999). The *Synechocystis* genome contains four genes encoding small proteins with a Cab-like domain (HliA-D). HliA, HliB and HliC have been detected in the CP47 module and later PSII assembly intermediates containing CP47 (Promnares et al. 2006; Yao et al.^2007^; Konert et al. 2021), while HliD together with HliC has been found in association with RCII (Knoppová et al. 2014) and Chl synthase (Chidgey et al. 2014). Although single *hlip* deletion mutants did not show apparent phenotypic differences from wild type (WT), a mutant lacking all Hlips is very sensitive to high light and oxidative stress (Havaux et al. 2003) and exhibits increased generation of reactive oxygen species as well as fast photodamage (Sinha et al. 2012), suggesting the crucial role of these proteins during acclimation to stress conditions. Their protective role has been supported by the analyses of isolated HliD and HliC that bind Chl and β-carotene (Knoppová et al. 2014; Shukla et al. 2018) and can efficiently quench Chl excitation via the S1 state of β-carotene (Staleva et al. 2015).

The *Synechocystis* genome also encodes a protein with an N-terminus remarkably similar to the N-termini of two members of the Hlip family, HliA and HliB. However, this protein, encoded by the *ssl1498* gene, does not contain the Cab domain, and therefore, it most probably does not bind Chl and it is not expected to quench Chl excitation. In cyanobacterium *Thermosynechococcus elongatus,* the homolog of this protein was recently detected in PSII assembly intermediates (*T. elongatus*; Zabred et al. 2021; Xiao et al. 2021) in which it may contribute, together with Psb28, to the prevention of light-induced oxidative damage to these complexes. This protein has been named Psb34 (Zabret et al. 2021).

In the current study, we identified the *Synechocystis* Psb34 protein as a component of the dimeric and monomeric PSII complex and RC47. We constructed a Psb34-less mutant that exhibits an increased accumulation of HliA/B under standard conditions, suggesting that the N-terminal sequence present in both Psb34 and HliA/B is important for their binding to CP47. Unlike HliA/B, Psb34 does not bind to the CP47 assembly module and data suggest that it is important for the balanced distribution and recycling of HliA/B among the individual CP47-containing PSII assembly intermediates. Co-isolation of tagged Psb34 with PSII complexes containing both assembly factors and OEEs suggests that Psb34 participates in the late stages of PSII biogenesis when the assembly intermediates are converted into the fully functional PSII complexes.

## Materials and methods

### Construction of mutant strains

The glucose-tolerant strain of *Synechocystis* sp. PCC 6803 GT-P (Tichý et al. 2016) was used as the control WT strain and all strains used in this study and derived from this WT are listed in Online resource Table S1.

The Psb34-less strain, ΔPsb34 was constructed by the replacement of the *psb34* gene with a zeocin antibiotic resistance cassette. To make an appropriate deletion construct, we applied the two-step megaprimer method (Ke and Madison 1997). In the first step, we amplified the sequences upstream and downstream (500 bp) of the *psb34* gene using fusion primers (Psb34-2Z and Psb34-3Z, Online resource Table S2) recognizing in one direction the *psb34* gene and the zeocin cassette in the other direction. The fusion primers were used in pairs with Psb34 forward and reverse primers (Online resource Table S2). In the second step, we amplified the zeocin resistance cassette (*Streptoalloteichus hindustanus*, Invitrogen) using PCR products from the first step as primers. Finally, for amplifying the complete deletion construct, we utilized the *psb34* forward and reverse primers. To construct the Psb34-less mutant strains, the cells of WT and other strains used were transformed with the final PCR product (Online resource Fig. S1). Mutants were segregated using a gradually increasing concentration of zeocin. Segregation was confirmed by PCR using Psb34-1Z and Psb34-4Z primers (Online resource Table S2).

Plasmid pPD-NFLAG was used for the construction of strains expressing FLAG-tagged Psb34 under the control of the *psbAII* promoter as described in Chidgey et al. (2014). The primers used are shown in Online resource Table S2. Subsequently, the construct was used for the transformation of ∆Psb34 cells. The mutant was selected for kanamycin resistance, segregated using a gradually increasing concentration of kanamycin and its full segregation was confirmed by PCR utilizing gene-specified primers (Online resource Table S2).

### Growth conditions

The strains were grown in liquid BG-11 medium under 40 µmol photons m^−2^ s^−1^ (normal light, NL) at 29 °C. Medium was supplemented with 5 mM glucose for non-autotrophic ∆CP47 and ∆CP43 mutants. For low-light (LL) and high-light (HL) treatments, the cells were first grown under NL conditions until the exponential phase (OD_750 nm_ of about 0.6–0.8). Subsequently, the cultures were transferred to 5 µmol photons m^−2^ s^−1^ (LL) or 500 μmol photons m^−2^ s^−1^ (HL).

For growth on agar plates, the liquid culture with cells in the exponential phase was diluted to OD_750 nm_ of 0.1, 0.01 and 0.001 and spotted on BG-11 agar plates containing BG-11 and 10 mM TES/NaOH, pH 8.0. For non-autotrophic strains, the agar additionally contained 5 mM glucose. Plates were exposed to LL, NL, HL or intermittent light (IL, 5 min 500 µmol photons m^−2^ s^−1^ and 5 min dark) for 8 days.

### Radioactive labelling

Radioactive pulse labelling of the cells was performed at 500 µmol photons m^−2^ s^−1^ and 30 °C using a mixture of [^35^S]Met and [^35^S]Cys (Hartmann Analytic Gmbh, Braunschweig, Germany) as described previously (Dobáková et al. 2009).

### Whole-cell absorption spectroscopy and pigment determination

Absorption spectra of whole cells were measured at room temperature using a UV-3000 spectrophotometer (Shimadzu, Japan) in cultures with the identical OD_750 nm_. For routine determination of Chl content, pigments were extracted from cell pellets with 100% methanol and Chl concentration was determined spectroscopically (Ritchie 2006).

### Low-temperature fluorescence spectroscopy

77 K Chl fluorescence emission spectra were measured in cultures with the identical OD_750 nm_ using an SM 9000 spectrophotometer (Photon Systems Instruments, Czech Republic) at an excitation wavelength of 455 nm, as described in (Kotabová et al. 2021). The spectra were normalized to the 570 nm maximum of rhodamine (1 µM) used as an internal standard.

### PSII activity

The activity of PSII was measured as the rate of oxygen evolution using a Clark-type electrode in the presence of the artificial electron acceptors *p*-benzoquinone (2.5 mM) and potassium ferricyanide (1 mM). The data are averages ± SD of three biological experiments, three repetitions in each.

### Preparation of membrane fraction and analysis of proteins

For small-scale preparation of cellular membranes, cells (40 ml) were harvested at OD_750nm_ ≈ 0.6–0.8, pelleted, washed, and resuspended in buffer A (25 mM MES/NaOH, pH 6.5, 10 mM CaCl_2_, 10 mM MgCl_2_, 25% glycerol). Cells were broken using zirconia–silica beads in a tissue homogenizer (Precellys Evolution, Bertin Instruments, France). The membrane and soluble fractions were separated by centrifugation at 36,000×*g* for 20 min. Afterwards, the membranes were resuspended in buffer A. After measurement of Chl concentration, isolated membranes were solubilized with β-dodecyl-maltoside (DDM, ratio DDM/Chl = 60 (w/w)) and then analysed using two-dimensional PAGE consisting of clear native (CN) PAGE in a 4–14% gradient gel (Komenda et al. 2019) and SDS-PAGE in a denaturing 16–20% gradient gel containing 7 M urea (2D-CN/SDS-PAGE). The standard one-dimensional SDS-PAGE was performed in the same 12–20% denaturing gel and membrane proteins were solubilized with 1% (w/v) SDS and 1% (w/v) DTT for 30 min at room temperature prior to loading onto the gel. For autoradiography, the gels were stained with Coomassie Blue (CBB), destained, dried and exposed to a Phosphoimager plate for 24 h. For the detection of proteins, the gels were stained with SYPRO Orange and subsequently transblotted to a polyvinylidene difluoride (PVDF) membrane. We used anti-rabbit antibodies specific to: D1, CP47 and CP43 (Komenda et al. 2008); PsaD (Xu et al. 1994) and HliA/B (cat. no. AS10 1603, Agrisera, Sweden). We also prepared a new anti-rabbit antibody against Psb34, specific to a peptide 25–36 of the *Synechocystis* protein. The blots were developed using the anti-rabbit secondary antibody conjugated with horseradish peroxidase (Sigma-Aldrich, USA) and chemiluminescence substrate Immobilon Crescendo (Merck, USA). Western blot analyses were repeated at least twice, autoradiograms three times, all with consistent results.

### FLAG-tag protein purification

For FLAG-tag protein purification, 2 L of cellculture were grown in 10L flasks under 40 µmol photons m^−2^ s^−1^ in BG-11 medium until they reached the exponential phase (OD_750nm_ ≈ 0.6–0.8) and then were exposed to 500 µmol photons m^−2^ s^−1^ for 2 h. The cell culture was agitated with a magnetic stirrer and bubbled with air. Cells were centrifuged, washed and resuspended in buffer A containing a protease inhibitor cocktail (Roche, USA, or Sigma-Aldrich, USA). Cells were broken using a tissue homogenizer (Precellys Evolution, Bertin Instruments, France). The membranes were solubilized with β-DDM and FLAG-tagged proteins were pulled down using the anti-FLAG M2 affinity gel (Sigma-Aldrich, USA) according to Koskela et al. (2020). The isolation was repeated three times with consistent results.

### Mass spectrometric analysis of isolated preparations

The proteins in whole isolated preparations were analysed after acetone precipitation. 50 µl of acetone cooled to − 20 °C were added to the whole protein fraction and after one hour of incubation at − 20 °C, the sample was spun down for 10 min at 20,000×*g* and 4 °C. Supernatant was removed and the rest of the acetone was evaporated under a fume hood for approx. 30 min. The precipitate was dissolved in 10 µl of 40 mM ammonium bicarbonate in 9% acetonitrile containing 0.4 mg trypsin (proteomics grade; Sigma-Aldrich) and incubated at 37 °C overnight. Excess liquid was removed by Speedvac, and 40 µl of solvent A (0.1% formic acid in water) was added to the 10 µl of tryptic digest. MS analysis was performed on a NanoElute UHPLC (Bruker) online coupled to the ESI Q-ToF a high-resolution mass spectrometer (Bruker Impact HD). Trapping was followed by a reverse-phase UHPLC two column separation, using the Thermo Trap Cartridge as a trap column and Bruker Fifteen C18 analytical column (75 mm i.d. 150 mm length, particle size 1.9 mm, reverse phase). The linear gradient elution ranged from 95% solvent A (0.1% formic acid in water) to 95% solvent B (0.1% formic acid in acetonitrile and water (90/10)) at a flow rate of 0.3 ml/min and time 60 min. Eluted peptides flowed directly into the ESI source. Raw data were acquired in the Dynamic MS/MS Spectra Acquisition with the following settings: dry temperature of 150 °C, drying gas flow of 3 l/min, capillary voltage of 1300 V and endplate offset of 500 V. The spectra were collected in the range 150–2000 m/z with spectra rate 2 Hz. The CID was set as a ramp from 20 to 60 eV on masses 200–1200, respectively. The acquired spectra were submitted for database search using the MaxQuant software against *Synechocystis* protein databases from the Uniprot Web site (https://www.uniprot.org/proteomes/UP000001425). N-terminal acetylation, deamidation of Asn and Gln, carbamidomethylation of Cys and oxidation of Met were set as variable modifications. Identification of three consecutive y-ions or b-ions was required for a positive peptide match.

## Results

### Psb34 associates with CP47-containing PSII complexes

**Fig. 1:**
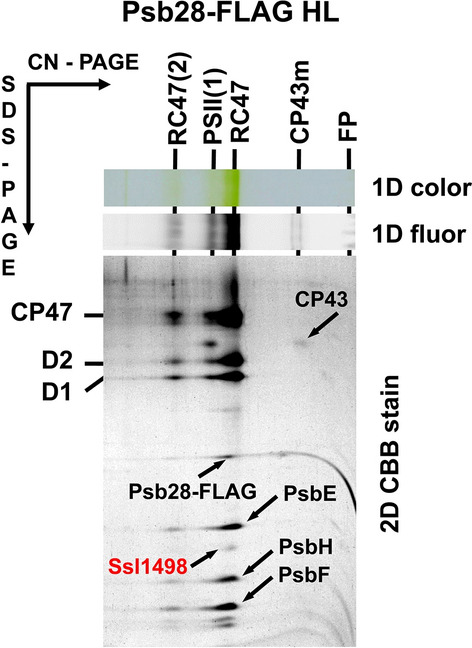
2D analysis of membrane proteins of the Psb28-FLAG preparation isolated from the strain expressing Psb28-FLAG. The preparation was analysed using 2D-CN/SDS-PAGE. After the first dimension, the gel was photographed (1D color) and scanned for Chl fluorescence (1D fluor). After separation in the second dimension, the 2D gel was stained using Coomassie Blue (2D CBB stain) and designated proteins were determined by MS. Designation of complexes: PSII(1), monomeric PSII core complexes; RC47(2) and RC47, the dimeric and monomeric PSII core complex lacking CP43; CP43, PSII antenna CP43 module; FP, free pigments. The loaded sample contained 0.5 µg of Chl

**Fig. 2:**
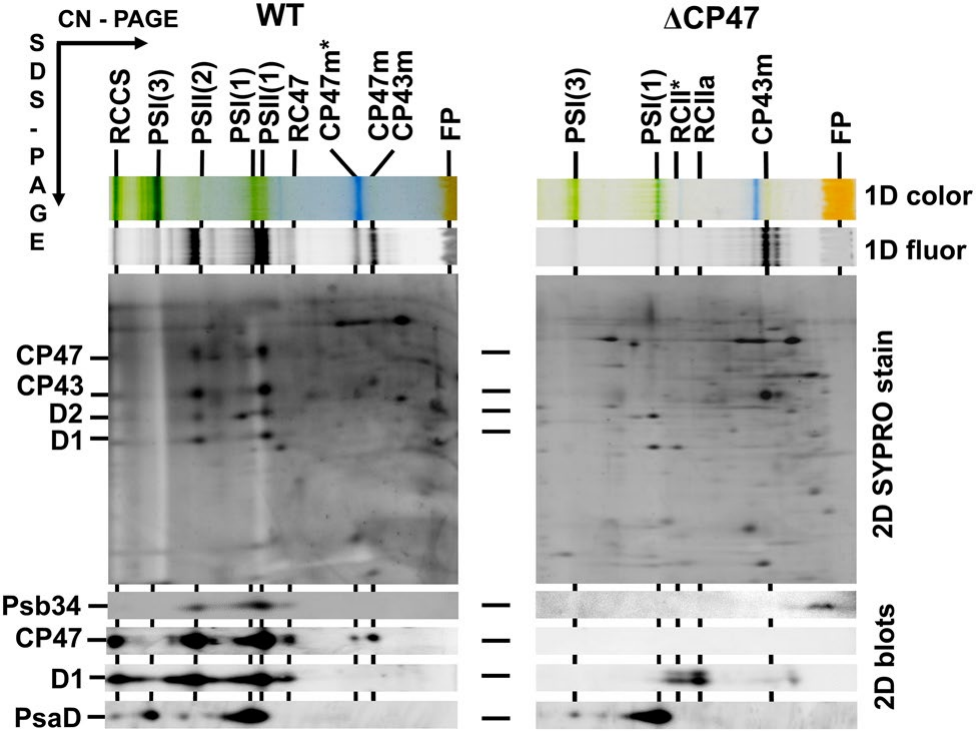
Identification of Psb34 protein in the membranes of WT and CP47-less (ΔCP47) strains. Membranes isolated from the strains were analysed using 2D-CN/SDS-PAGE. After the first dimension, the gel was photographed (1D color) and scanned for Chl fluorescence (1D fluor). After separation in the second dimension, the 2D gel was stained using SYPRO Orange (2D SYPRO stain), blotted to a PVDF membrane (2D blots), and Psb34, CP47, D1 and PsaD were detected by specific antibodies. Designation of complexes: RCCS, supercomplex of PSI and PSII; PSI(3) and PSI(1), trimeric and monomeric Photosystem I; PSII(2), dimeric PSII core complex; RCII* and RCIIa, PSII complexes lacking CP43 and CP47; CP47m*, CP47 module containing Psb35; CP47m and CP43m, unassembled CP47 and CP43 modules; other designations as described in Fig. 1. Each loaded sample contained 5 µg of Chl

Detailed analysis of complexes previously isolated using the FLAG-tagged PSII assembly factor Psb28 (Bečková et al. 2017) revealed the presence of a protein, the homologue of which (encoded by the *tsl0063* gene) was recently identified in PSII assembly intermediate complexes of *T. elongatus* (Zabret et al. 2021; Xiao et al. 2021) and named Psb34. In *Synechocystis*, this protein is encoded by the *ssl1498* gene and in the C-terminal FLAG-tagged Psb28 (Psb28-FLAG) preparation analysed using 2D-CN/SDS-PAGE, it was identified as a protein of about 9 kDa located in a PSII assembly intermediate RC47 (Fig. 1). We prepared a specific antibody against it to test for the presence of Psb34 in membrane complexes of WT cells (Fig. 2). We confirmed that the protein is PSII-specific, as we detected it in PSII(2), PSII(1) and RC47 (Fig. 2). We were also interested in whether Psb34 binds to the RCII complex lacking CP47 and CP43 (Komenda et al. 2004). Therefore, membranes of a CP47-less strain accumulating RCII (Komenda et al. 2004) were also assessed for the presence of Psb34. The protein was missing in RCII and only a small amount of Psb34 was detected in the region of unassembled proteins (Fig. 2), so the interaction of Psb34 with PSII is dependent on the presence of CP47.

The N-terminal amino acid sequence of Psb34 shows a remarkable similarity to that of HliA/B members of the Hlip family (Fig. S2). Since HliA/B associates with CP47-containing complexes in a PsbH-dependent manner (Promnares et al. 2006), we also tested the ability of Psb34 to bind PSII complexes in a mutant lacking PsbH. Indeed, in the absence of this small PSII protein, Psb34 was not present in PSII complexes, and we only detected it in the region of unassembled proteins using 2D-CN/SDS-PAGE (Fig. S3).

**Fig. 3:**
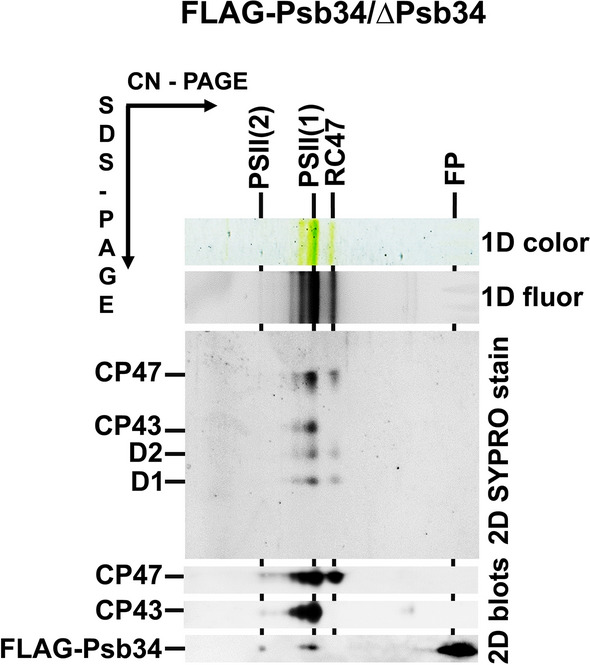
2D analysis of membrane proteins of the FLAG-Psb34 preparation isolated from the strain expressing FLAG-tagged Psb34. The preparation was analysed using 2D-CN/SDS-PAGE. After the first dimension, the gel was photographed (1D color) and scanned for Chl fluorescence (1D fluor). After separation in the second dimension, the 2D gel was stained using SYPRO Orange (2D SYPRO stain), blotted to a PVDF membrane (2D blots) and CP47, CP43 and FLAG-Psb34 were detected by specific antibodies. Designation of complexes is described in Figs. 1 and 2. The loaded sample contained 0.5 µg of Chl

To further confirm the association of Psb34 with PSII complexes, we constructed a strain lacking the original Psb34 but expressing its N-terminally FLAG-tagged variant under the *psbA2* promotor. Purification of the FLAG-tagged Psb34 from detergent-solubilized mutant membranes using a FLAG-specific immunoaffinity pull-down resulted in co-isolation of PSII(2), PSII(1) and RC47 as shown using 2D-CN/SDS–PAGE (Fig. 3). Analysis of the pull-down using mass spectrometry revealed the presence of PSII auxiliary factors that assist PSII biogenesis such as Psb27 and Psb28, but PsbO and PsbV, subunits stabilizing the oxygen-evolving CaMn_4_O_5_ cluster, were also detected in the preparation (Table 1). Interestingly, no Hlips were found in the preparation although it was isolated from membranes of high light-exposed cells, suggesting that binding of Psb34 and HliA/B is mutually exclusive.

**Table 1:** List of proteins identified by mass spectrometry of the FLAG-Psb34 pull-down isolated from FLAG-Psb34/∆Psb34 cells exposed to high light (500 µmol photons m^−2^ s^−1^) for 2 h

Protein, ID	Molecular weight (kDa)	Length	Coverage (%)	Number of peptides	PLGS score^a^
CP47 P05429	55,902	507	40.8	20	311
CP43 P09193	50,302	460	39.3	17	199
D2 P09192	39,490	352	261	7	79
D1 P16033	39,721	360	20.3	6	80
Psb28 Q55356	12,590	112	75	6	68
Psb27 P74367	14,780	134	49.3	5	56
PsbH P14835	7110	64	21.9	1	15
PsbO P10549	29,911	274	7.3	1	10
PsbV Q55013	17,884	160	11.9	1	6

^a^PLGS score is calculated by Protein Lynx Global Server (PLGS 2.2.3) software (Waters) and is a statistical measure of peptide assignment accuracy

### The Psb34-less mutant shows no apparent phenotypic distinction from WT

**Fig. 4:**
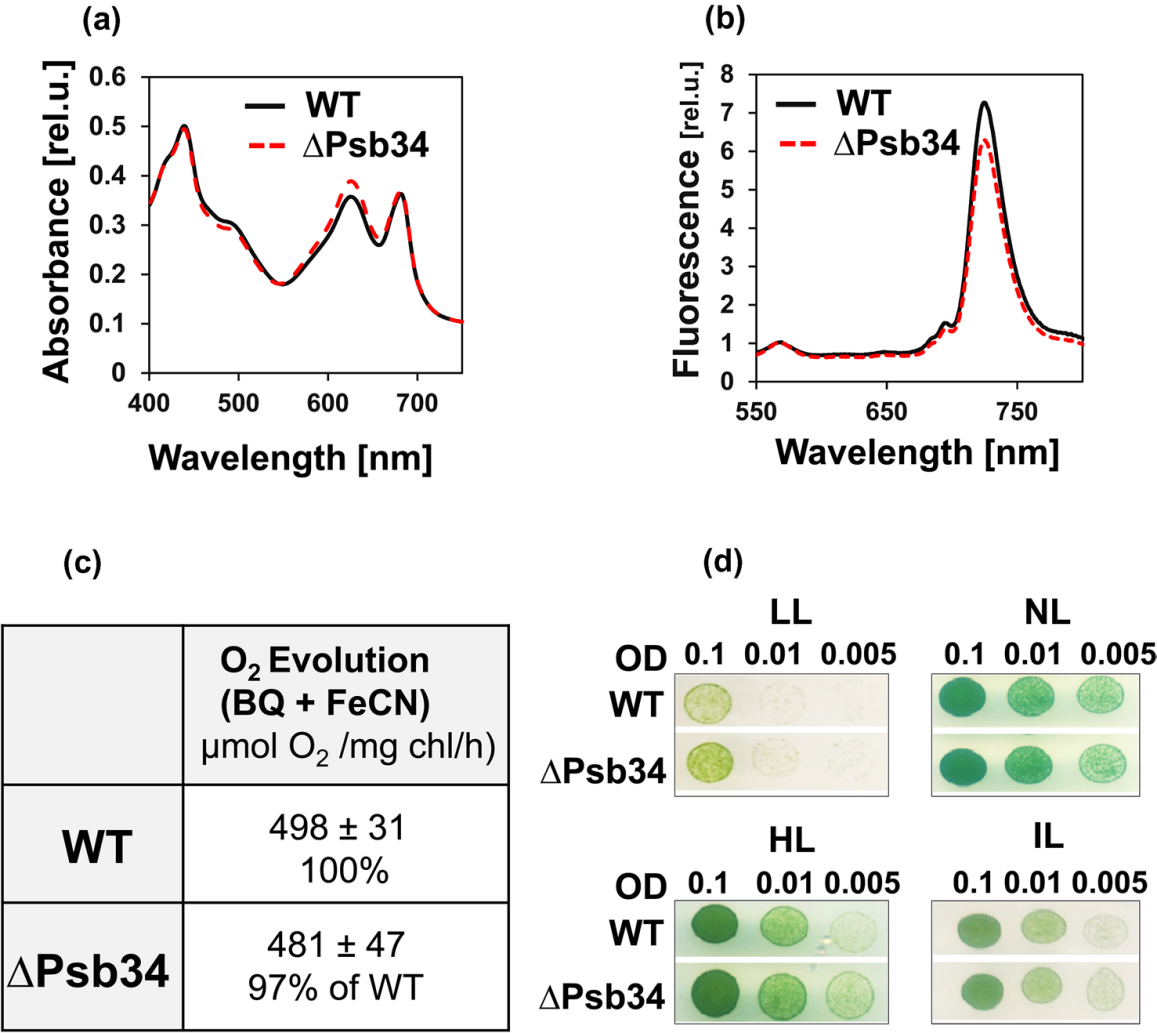
Whole-cell absorption spectra (**a**), 77 K Chl fluorescence spectra (**b**), PSII-mediated oxygen evolution (**c**) and growth (**d**) of WT and ΔPsb34 strains. **a** Whole-cell absorption spectra of WT (black solid line) and ΔPsb34 (red dashed line) liquid cultures are shown after normalization to the optical densities at 750 nm. **b** For 77 K fluorescence spectra equal amounts of cells from WT (black solid line) and ΔPsb34 (red dashed line) liquid cultures were frozen in liquid nitrogen and excited at 435 nm; spectra were normalized to the emission peak of the internal standard rhodamine at 570 nm. **c** The light-saturated rate of oxygen evolution in the presence of 2.5 mM p-benzoquinone and 2 mM potassium ferricyanide was measured in the exponentially grown cultures using a Clark electrode; values represent means of three biological replicates and three measurements each ± SD. **d** Cells of WT and ΔPsb34 were spotted on the agar plates containing BG-11 and 10 mM TES/NaOH, pH 8.0 and grown under LL, NL, HL or intermittent light (IL, 5 min 500 µmol photons m^−2^ s^−1^/5 min dark) for 8 days

**Fig. 5:**
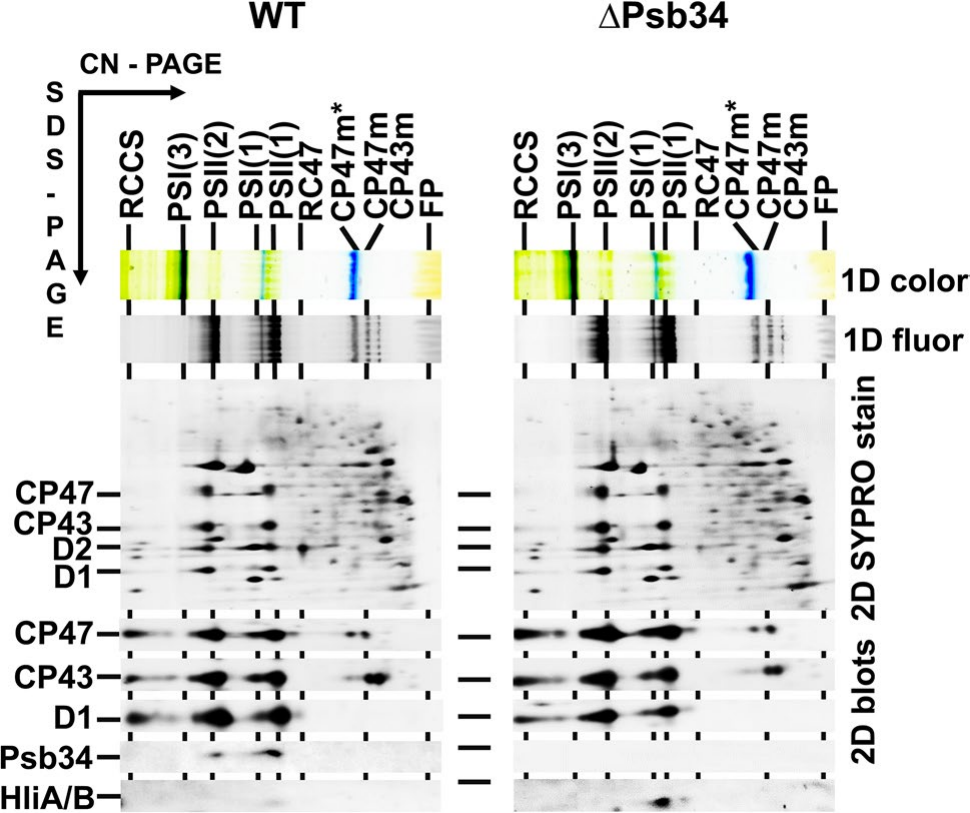
2D analysis of membrane proteins of WT and ΔPsb34. Membranes isolated from the strains grown under NL were analysed using 2D-CN/SDS-PAGE. After the first dimension, the gel was photographed (1D color) and scanned for Chl fluorescence (1D fluor). After separation in the second dimension, the 2D gel was stained using SYPRO Orange (2D SYPRO stain), blotted to a PVDF membrane (2D blots) and CP47, CP43, D1, Psb34 and HliA/B were detected by specific antibodies. Designation of complexes is described in Fig. 2. Each loaded sample contained 5 µg of Chl

To define the function of Psb34 protein, we constructed a mutant in which the *psb34* gene was replaced with a zeocin resistance cassette, and we compared it with WT. Under standard growth conditions, ΔPsb34 cells did not show any remarkable difference from WT in pigmentation (Fig. 4a), PSII/PSI ratio (Fig. 4b) or oxygen-evolving activity (Fig. 4c). Moreover, growth under normal, low, high and intermittent light conditions was also very similar between WT and ΔPsb34 cells (Fig. 4d). We also analysed membranes isolated from both strains using 2D-CN/SDS-PAGE to compare the pattern of membrane complexes between strains. Native gel, staining of the proteins in the 2D gel, and subsequent immunoblotting using antibodies specific to large PSII proteins did not reveal any apparent difference between WT and ΔPsb34 (Fig. 5). However, immunodetection of Psb34 confirmed the absence of this protein in ΔPsb34 (Fig. 5) and the HliA/B proteins, which are undetectable in PSII complexes of WT under standard growth conditions, were clearly observed in PSII(1) of the mutant cells. This result indicates that Psb34 and HliA/B bind to the same or a similar site within PSII and that in its absence HliA/B can bind to PSII complexes even in unstressed cells. An alternative explanation is that the absence of Psb34 causes oxidative stress (Zabred et al. 2021), which leads to activation of HliA/B gene transcription and subsequently to increased HliA/B protein levels even under NL conditions. However, our analysis of HliA/B transcripts revealed comparable HliA/B transcript levels in both strains, which cannot explain the appearance of HliA/B in PSII(1) in WT cells under standard conditions.

**Fig. 6:**
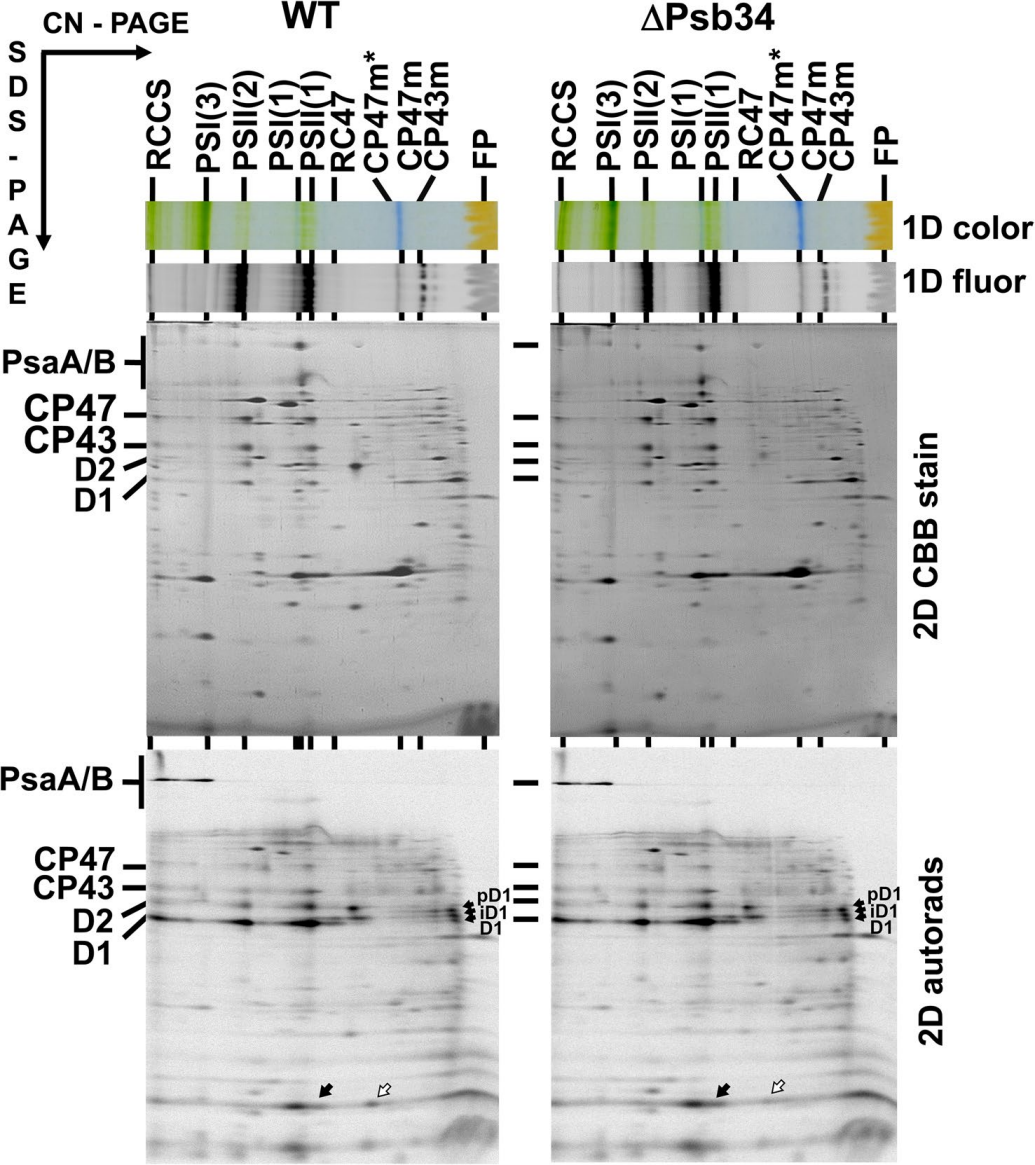
2D analysis of radioactively labelled membrane proteins of WT and ∆Psb34 autotrophic strains. Membranes isolated from radioactively labelled cells were analysed using 2D-CN/SDS-PAGE. After the first dimension, the gel was photographed (1D color) and scanned for Chl fluorescence (1D fluor). After separation in the second dimension, the 2D gel was stained with Coomassie Blue (2D CBB stain) and the radiolabelled proteins were subsequently detected by autoradiography (2D autorads). Designation of complexes as described in Fig. S3; RC47 is PSII core complex lacking CP43; closed arrows designate different unassembled D1 forms and in the lower part of the autoradiogram HliA/B in PSII(1) and RC47; the empty arrows designate HliA/B in CP47m. Each loaded sample contained 5 µg of Chl

We also monitored the synthesis of membrane proteins in WT and ΔPsb34 mutant cells by radioactive labelling using a mixture of [^35^S]Met/Cys. Membranes isolated from these cells were analysed by 2D-CN/SDS-PAGE and autoradiography (Fig. 6). Labelling of the main PSI and PSII proteins including HliA/B was very similar in both strains, but there was a difference in the distribution of labelled HliA/B proteins among PSII complexes. When compared with WT, HliA/B in ΔPsb34 were labelled less in CP47m and more in PSII(1) and RC47. This indicates that Psb34, which does not interact with CP47m, could be important for the sufficient association of HliA/B with CP47m by limiting their binding to PSII(1) and RC47.

### Accumulation of HliA/B and Psb34 in WT and ∆Psb34 cells exposed to high irradiance

**Fig. 7:**
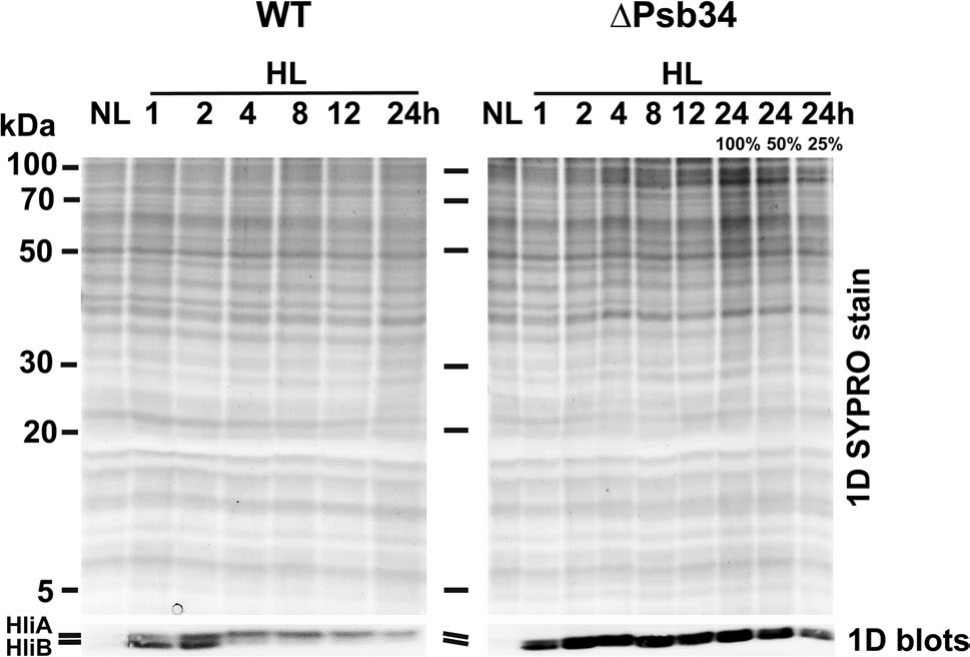
1D analysis of the thylakoid membrane proteins of WT and ∆Psb34 exposed to NL and to HL (500 μmol photons m^−2^ s^−1^) for 1, 2, 4, 8, 12 and 24 h. Membranes isolated from the strains were analysed using SDS-PAGE and 1D gel was stained with SYPRO Orange (1D SYPRO stain) and blotted onto a PVDF membrane (1D blots) and HliA/B were detected with specific antibody. The upper line in 1D blot belongs to HliA and the lower line belongs to HliB. SYPRO-stained gel shows equal loading of the samples. Each loaded sample contained 2 µg of Chl (100%)

The physiological role of HliA/B has yet to be fully clarified, although it is assumed that they protect their associated PSII complexes from photodamage (Komenda and Sobotka 2016) and in this way contribute to the acclimation of cyanobacteria to high light and other stresses. Accumulation of His-tagged HliA and HliB under high irradiance has been individually followed (He et al. 2001), but this analysis has not allowed for their parallel detection and a direct comparison of their levels. Since our 1D gel system allows for parallel detection, and as HliA has a slightly lower mobility than HliB (Yao et al. 2007; Konert et al. 2021), we followed the accumulation of HliA and HliB during 24-h HL treatment (Fig. 7). The Western blot analysis showed that accumulation of HliB in WT was highest during the first 2 h of illumination when its level largely exceeded the level of HliA. Later, its amount dropped way down and became barely detectable after 8 h of illumination. HliA reached its maximum after 2 h, then its level slowly decreased over time. Nevertheless, after 24 h of high irradiance, its signal remained apparent. In ΔPsb34, the overall level of both Hlips was markedly higher compared with WT, and levels of HliA and HliB remained high over the entire 24-h period (Fig. 7). Data suggest that HliA is especially important during long-term stress, while HliB seems to play a main role during the initial stage of HL treatment. The significant decrease in HliA/B levels during HL treatment in WT contrasted with a high and stable level of HliA/B in ΔPsb34. The amount of Psb34 in WT quickly decreased, and after 2 h, it became undetectable (Fig. S4), again confirming the inverse relationship between Psb34 and HliA/B.

**Fig. 8:**
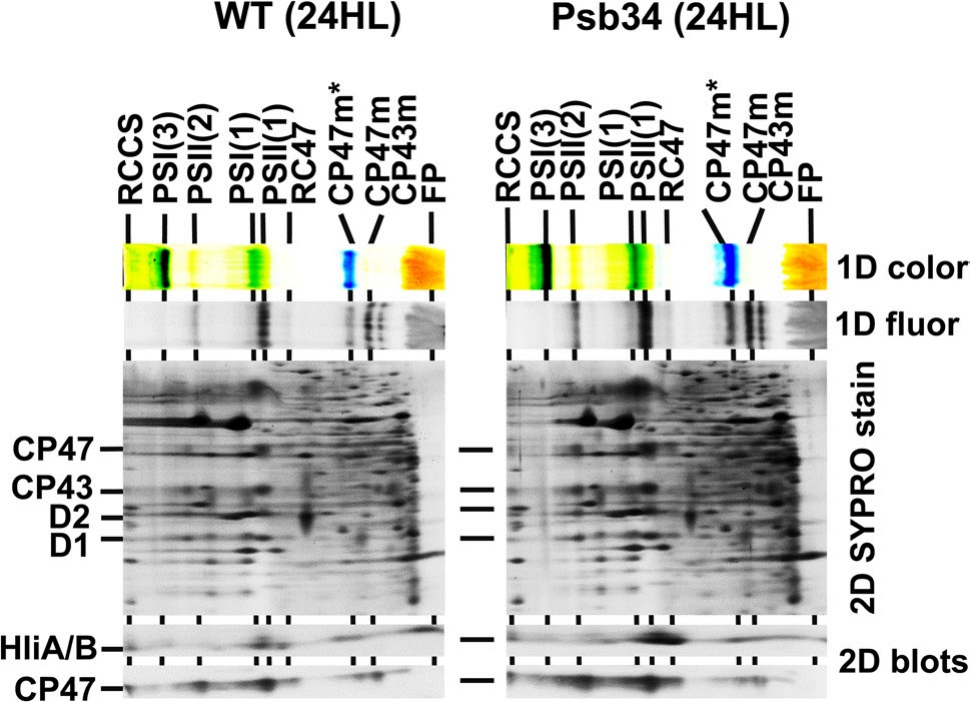
2D analysis of membrane proteins from WT and ∆Psb34 strains exposed to HL for 24 h. Membranes isolated from the strains were analysed using 2D-CN/SDS-PAGE. After the first dimension, the gel was photographed (1D color) and scanned for Chl fluorescence (1D fluor). After separation in the second dimension, the 2D gel was stained with SYPRO Orange (2D SYPRO stain) and blotted onto a PVDF membrane (2D blots), and HliA and CP47 were detected with specific antibodies. Designation of complexes as described in Fig. 2. Each loaded sample contained 5 µg of Chl

To obtain more complete information about the distribution of HliA/B among PSII complexes in HL-treated cells, we analysed membranes isolated from WT and ∆Psb34 cells exposed to HL for 24 h using 2D-CN/SDS-PAGE. This analysis confirmed a generally higher HliA/B content in cells of ∆Psb34 when compared with WT. These proteins were also much more abundant in PSII(1), especially in RC47, than in CP47m in ∆Psb34 cells, while in WT, the distribution was more homogenous (Fig. 8). Since in 2D gels we were not able to separate HliA and HliB, we assessed their individual content in PSII complexes by analysing HL-exposed HliA- and HliB-less strains. In agreement with WT 1D gel data (Fig. 7), the 2D gel in combination with immunoblotting showed a low accumulation of HliB in PSII(1), RC47 and CP47m in the absence of HliA after 24 h of HL, and this was also accompanied by decreases in the amounts of PSII(1), PSII(2) and Psb34. In the absence of HliB, the level of HliA in PSII complexes increased during HL treatment and the levels of PSII complexes and Psb34 remained rather stable (Fig. S5a).

To see a possible effect of Psb34 on the accumulation of individual HliA and HliB, we additionally deleted the *ssl1498* gene in the HliA- and HliB-less mutants. The resulting ∆HliA/∆Psb34 and ∆HliB/∆Psb34 double mutants were again exposed to HL and the levels of HliA, HliB and PSII were monitored after 24 h of HL treatment (Fig. S5b). Based on our previous results obtained with WT and the ∆HliA single mutant, we expected a decreasing amount of HliB in the ∆HliA/∆Psb34 double mutant after 24 h of HL exposure. However, we found HliB accumulating in the ∆HliA/∆Psb34 strain even after 24 h of HL (Fig. S5b). This result suggested that the absence of Psb34 facilitated binding of HliB to PSII and allowed accumulation of HliB over the entire 24-h period. On the other hand, the high accumulation of HliA in ∆HliB/∆Psb34 cells after 24-h HL treatment (Fig. S5b) was consistent with the results obtained using the single HliB-null mutant (Fig. S5a). In both double mutants, Hlips were present in PSII(1) and as unassembled proteins but were absent in CP47m, suggesting that Psb34 might promote the HliA/B binding to CP47m via the detachment of part of HliA/B from PSII(1). Overall, the data showed that HliA is a more stable protein, important during long-term acclimation of cells to stress, while the level of HliB is more variable, quickly responding to stress. HliB, compared to HliA, is also more affected by the absence of Psb34. Interestingly, the ∆Psb34 strain lacking HliA was the only Psb34-less mutant which showed slower growth under an intermittent light regime, while the growth of ∆Psb34 and ∆HliB/∆Psb34 mutants did not differ from WT in any light regime (Fig. S6).

### In the CP43-less strain HliA/B extremely overaccumulate when Psb34 is absent

**Fig. 9:**
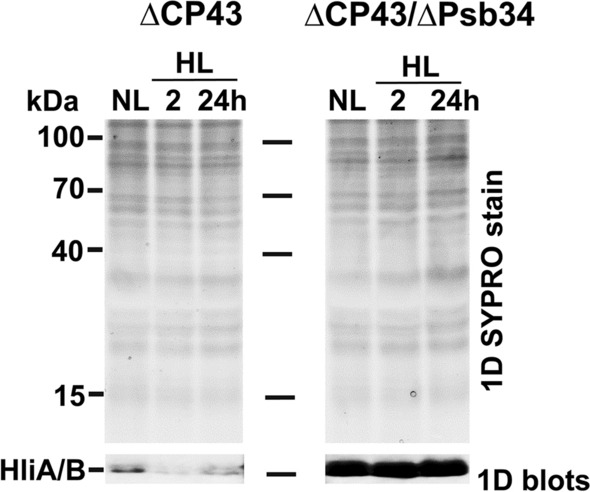
1D analysis of the thylakoid membrane proteins of ∆CP43 and ∆CP43/∆Psb34 exposed to NL and to HL conditions. NL: normal light (40 µmol photons m^−2^ s^−1^) HL: high light (500 µmol photons m^−2^ s^−1^). The 1D gel was stained with SYPRO Orange (2D SYPRO stain) and blotted onto a PVDF membrane (1D blots), and HliA/B were detected with specific antibodies. SYPRO-stained gel shows equal loading of the samples. Each loaded sample contained 2 µg of Chl

Previous data showed that HliA/B and Psb34 were bound mostly in PSII(1), while their binding to RC47 was hardly visible due to the absence of RC47 in a majority of the strains (Figs. 2, 5 and S5). In order to judge whether all three proteins also bind to RC47, we analysed cells lacking CP43 alone or CP43 together with Psb34, HliA and HliB. It has been shown that removal of CP43 protein from *Synechocystis* cells leads to accumulation of the RC47 complex (Komenda et al. 2004), which also contains HliA/B even under standard growth conditions (Boehm et al. 2012). Western blot analysis of membranes isolated from the ∆CP43 single mutant and the ∆CP43/∆Psb34 double mutant under different conditions (NL, 2 h of HL and 24 h of HL) indicated a decreased amount of HliA/B upon light stress in ∆CP43 but their strong overaccumulation under both NL and HL conditions in ∆CP43/∆Psb34 (Fig. 9).

**Fig. 10:**
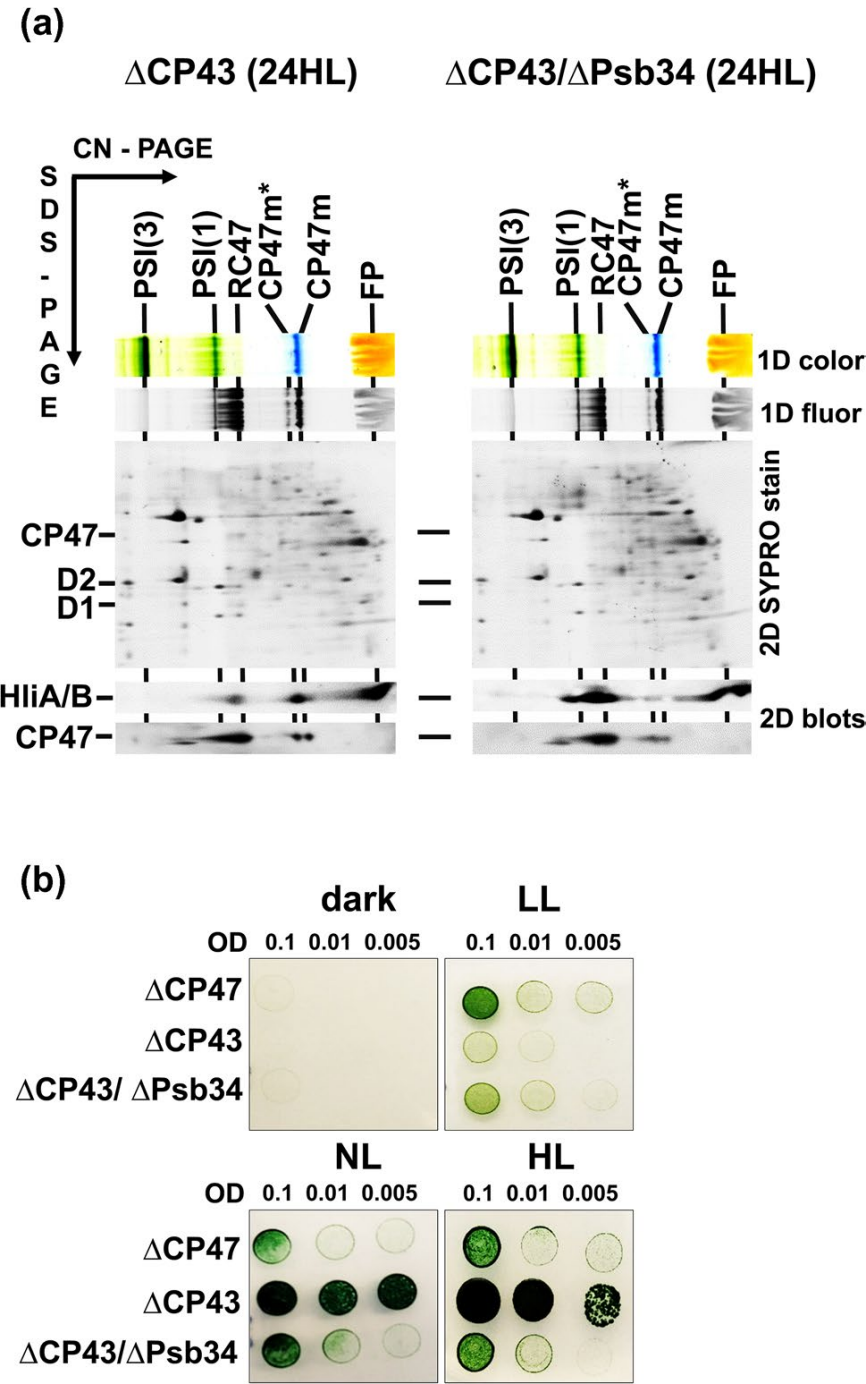
2D analysis of membrane proteins from ∆CP43 and ∆CP43/∆Psb34 strains exposed to HL for 24 h (**a**) and their growth in comparison with the CP47-less strain (**b**). **a** Membranes isolated from the strains were analysed using 2D-CN/SDS-PAGE. After the first dimension, the gel was photographed (1D color) and scanned for Chl fluorescence (1D fluor). After separation in the second dimension, the 2D gel was stained with SYPRO Orange (2D SYPRO stain) and blotted onto a PVDF membrane (2D blots), and HliA/B and CP47 were detected with specific antibodies. Designation of complexes is described in Fig. 2. Each loaded sample contained 3.5 µg of Chl. **b** Cells of ΔCP47, ΔCP43 and ΔCP43/ΔPsb34 were spotted on the agar plates containing BG-11, 10 mM TES/NaOH, pH 8.0 and 5 mM glucose and grown in the dark and under LL, NL and HL for 8 days

We also analysed membranes of ∆CP43 and ∆CP43/∆Psb34 mutants after 24 h of HL using 2D-CN/SDS-PAGE to see the distribution of HliA/B among PSII complexes. In ∆CP43 cells, HliA/B were present in RC47 and CP47m in approximately the same amounts (Fig. 10a). In contrast, in the ∆CP43/∆Psb34 strain, HliA/B were much more abundant in RC47 than in CP47m. Since Psb34 is not able to bind to CP47m, the data again support the hypothesis that Psb34 mediates an optimal equilibrium between the HliA/B content in RC47 and CP47m, and in this way may provide better photoprotection to CP47m. Also, the level of RC47 was lower and the CP47 signal belonging to CP47m was rather smeared in ∆CP43/∆Psb34 when compared to ∆CP43 indicating an oxidative damage. In agreement with these data, ∆CP43/∆Psb34 showed slower growth under high irradiance in comparison with ∆CP43 (Fig. 10b). Interestingly, in LL, the double mutant grew better than ∆CP43 alone. To find further support for the facilitated binding of HliB to RC47 in the absence of Psb34, we constructed ∆CP43/∆HliA/∆Psb34 and ∆CP43/∆HliB/∆Psb34 triple mutants and analysed their HliA/B distribution among PSII complexes after 24-h HL exposure using 2D-CN/SDS–PAGE. In the ∆CP43/∆HliA/∆Psb34 strain, HliB was only detected in the RC47 intermediate (Fig. S7a). This contrasted with the results of a similar experiment using the double ∆CP43/∆HliA mutant, in which a drastic decrease in HliB level in RC47 was detected after 24 h HL treatment (Fig. S7b). In the ∆CP43/∆HliB/∆Psb34 triple mutant, HliA also overaccumulated after 24 h HL exposure (Fig. S7a), but this was in agreement with the results using the ∆CP43/∆HliB double mutant (Fig. S7b). This strongly suggests that removal of Psb34 affects the amount of HliA in the RC47 complex much less than that of HliB. In both triple mutants, there were no HliA/B associated with CP47m, again supporting the importance of Psb34 for HliA/B binding to CP47m.

## Discussion

In the present study, we investigated a protein designated Psb34, a homologue of which was recently identified within the Psb28-containing PSII assembly complexes of *T. elongatus* (Zabret et al. 2021; Xiao et al. 2021). Initially, we detected Psb34 in purified Psb28-FLAG-containing RC47 (Fig. 1). This protein exhibits high similarity to the HliA and HliB members of the Hlip family at its N-terminus, but completely lacks the Chl binding motif in its transmembrane helix, which indicates that the function of Psb34 differs from HliA/B. We detected the protein in PSII(2), PSII(1) and RC47 but not in RCII and CP47m. This indicates that Psb34 and HliA/B bind to the same or a similar site on CP47 incorporated within PSII. In agreement with this, the binding of Psb34 to PSII complexes is inhibited or weakened in the absence of PsbH, a CP47-associated small PSII protein (Fig. S3), and this is also valid for HliA/B (Promnares et al. 2006). A common binding site for Psb34 and HliA/B was also strongly supported by co-isolation of the tagged Psb34 and HliA/B with a similar spectrum of CP47-containing PSII complexes. However, FLAG-Psb34 never co-isolated with HliA/B, even when isolated from HL-exposed cells (Table 1), and Psb34 was never detected in the His-HliA/B preparations (Konert et al. 2021). In addition, the His-HliA/B preparations contained the recently described Psb35 protein-stabilizing Hlips (Konert et al. 2021; Pascual Aznar et al. 2021), while FLAG-Psb34 preparations were free of this protein. This result also indicates that the additional helix observed in the PSII assembly intermediate from *T. elongatus* in proximity to the Psb34 homologue is neither Psb35 nor HliA/B (Xiao et al. 2021). It has been shown that the N-terminal sequence of Psb34 notably contributes to its binding to PSII assembly intermediates (Zabret et al. 2021; Xiao et al. 2021). Its high similarity to the sequence of HliA/B suggests the competitive binding of both proteins to the same binding site formed by the stromal exposed residues of CP47, D1, PsbH and PsbL (Online resource Fig. S2). Nevertheless, until the ultimate evidence for it is found based on a high-resolution structure of PSII complexes with bound HliA/B, we cannot completely exclude the possibility that HliA/B bind to a different site and this binding modifies the binding site of Psb34.

Similar to His-tagged HliA/B preparations, FLAG-Psb34 also co-isolated with Psb27 and Psb28 assembly factors (Konert et al. 2021), suggesting the involvement of Psb34 and HliA/B in PSII biogenesis. Conversely, in FLAG-Psb34, we identified OEE proteins PsbO and PsbV, while the His-HliA/B preparations were free of these proteins (Konert et al. 2021). This suggests that final conversion of assembly intermediates into the functional PSII complexes requires the removal of Hlips and Psb34 may play an important role in this process. In *T. elongatus*, deletion of the *psbJ* gene leads to a massive accumulation of an intermediate monomeric PSII complex containing the assembly factors Psb27, Psb28 and Tsl0063 (Zabret et al. 2021). Thus, this characterized complex also seems to be an assembly intermediate preceding PsbJ attachment and its associated activation of the PSII acceptor side during late stage of PSII biogenesis.

The distinct kinetics of HliA and HliB accumulation and their different distribution among PSII complexes during 24-h HL exposure suggest that they are not redundant interchangeable copies, rather each has its specific role during HL acclimation. Analyses of WT, HliA-less and HliB-less cells showed that after 24-h HL treatment, the level of HliB in PSII(1), RC47, CP47m and in the unassembled fraction markedly decreases (Figs. 7 and S5a). In contrast, HliA is more stably maintained during the whole 24-h period, although it is mainly present in PSII(1), RC47 and the unassembled fraction while its content in CP47m remains low (Fig. S5a). This is consistent with previous data showing limited binding of HliA to CP47m in the strain expressing His-tagged HliB (Komenda and Sobotka 2016) and with the low amount of CP47m co-isolated with His-HliA (Konert et al. 2021). Upon a sudden increase in irradiance, the immediate photoprotection of the newly made CP47 module might be crucial before Chl biosynthesis becomes attenuated and de novo Chl-dependent synthesis of CP47 is inhibited (Hollingshead et al. 2016). In this moment, the association of HliB with CP47m is essential for sustaining de novo biogenesis of PSII complexes that replace the extensively damaged ones. Later, when other acclimation mechanisms like the accumulation of xanthophylls and specific lipids (Zakar et al. 2017) or an increase in the level of FtsH proteases for faster PSII repair (Kopečná et al. 2012) are effectively induced, CP47m formation is less endangered and the presence of HliA is sufficient. Given the preference of HliA for PSII(1) and RC47, which are transiently formed during PSII repair (Komenda and Masojídek 1995; for reviews see Komenda et al. 2012a, b; Theis and Schroda 2016), HliA may be more specifically involved in PSII repair. In the CP43-less strain with extremely fast turnover of D1 (Komenda et al. 2006), HliA/B are present even without being exposed to stress conditions, but their level is surprisingly suppressed when the strain is subjected to excess light (Fig. 9). However, their low level might be partly related to the overall decreased RC47 content, which is most probably caused by the extremely fast photodamage resulting from the absence of electron donation from OEC.

Additional differences in the distribution of HliA and HliB among PSII complexes were induced by the removal of Psb34. The main difference was observed in ∆HliA/∆Psb34, in which HliB still accumulated in PSII complexes after 24 h of high light (Fig. S5b) although it was almost absent in the single ∆HliA mutant (Fig. S5a). This suggests that Psb34 preferentially regulates HliB binding to PSII assembly intermediates, while HliA seems to be more independent. Likewise, Psb34 maintained a stable level in PSII(1), independent of the amount of HliA, in the strain lacking HliB during high-light treatment, lending further support to the inverse relationship between Psb34 and HliB (Fig. S5b). The absence of Psb34 in the CP43 null mutants also resulted in the overaccumulation of HliA/B in the RC47 complex and again more HliB than HliA appeared in the double mutant (Fig. 9).

**Fig. 11:**
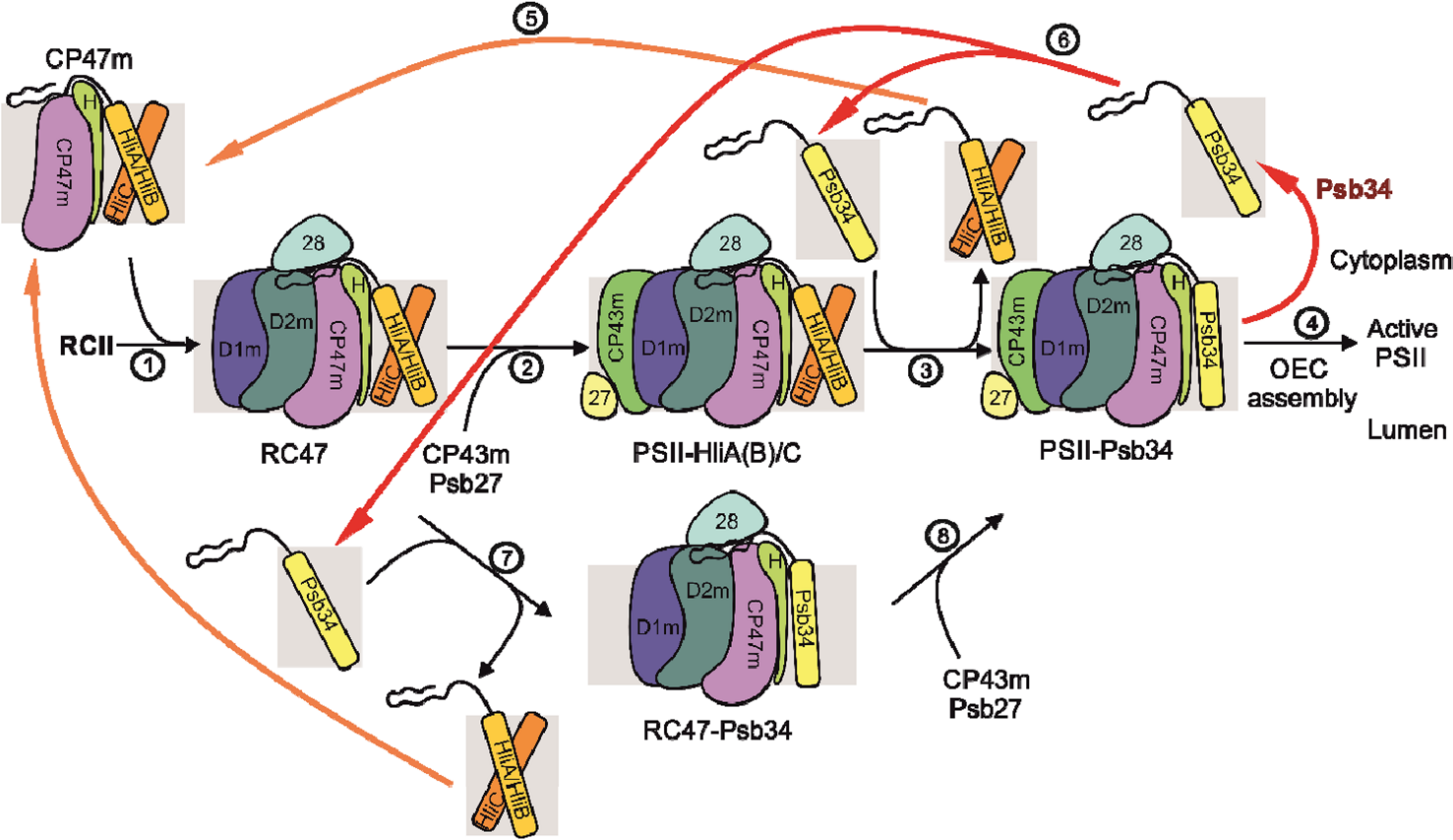
Model for the role of Psb34 under HL conditions. The PSII complex is formed by a stepwise process combining four building blocks called modules (D1m, D2m, CP43m, CP47m), which bind small PSII subunits and are pre-loaded with Chl and Car cofactors before association to larger PSII assembly intermediates (Komenda et al. 2012b). The formation of RCII complexes from D1m and D2m is followed by attachment of CP47m (step 1), forming the PSII core lacking CP43 (RC47). Under HL conditions, CP47m binds the HliA(B)/C pair which is then retained in RC47 (RC47-HliA(B)/C) and also in the monomeric PSII core complex (PSII-HliA(B)/C) formed after the attachment of CP43m (step 2). In PSII-HliA(B)/C, the Psb34 factor can replace the HliA(B)/C pair forming PSII-Psb34 (step 3). Psb34 is then released during the final conversion of the PSII-Psb34 intermediate into the active PSII (step 4). The released HliA(B)/C can be recycled and bind to another newly formed CP47m (step 5). Likewise, the released Psb34 can be reused (step 6), not only at the stage of PSII, but it can also replace HliA(B)/C from RC47-HliA(B)/C (step 7), which is then converted into PSII-Psb34 (step 8). Psb34 likely occupies the same binding site (Zabret et al. 2021; Xiao et al. 2021) as HliA(B)/C and this enables Psb34 to facilitate the detachment of HliA(B)/C from RC47 and PSII, keeping its amount sufficient for binding and photoprotection of CP47m. For simplification, the models of assembly intermediates do not contain most of the assembly factors and small PSII subunits, except PsbH (H), which is important for the binding of Psb34 and HliA(B)/C, and the Psb27 (27) and Psb28 (28) assembly factors that were identified in our FLAG-Psb34 pull-downs

An interesting difference between Psb34 and HliA/B is that the former does not interact/co-isolate with CP47m. This is rather puzzling when we take into consideration that the N-terminal amino acid residues involved in the interaction with CP47, PsbL and PsbH (Xiao et al. 2021) are conserved between Psb34 and HliA/B (Fig. S2). Although we expect that binding of HliA/B to CP47 is very similar to that observed for the *T.* *elongatus* homologue Tsl0063 (Zabret et al. 2021; Xiao et al. 2021), one factor possibly contributing to the stability of HliA/B binding to CP47m is their formation of a heterodimer with another member of the Hlip family, HliC (Konert et al. 2021). The contrast between HliA/B, which is able to stably bind CP47m, and Psb34, which is unable to do so, indicates that another function of Psb34 may be to limit binding of HliA/B to RC47 and PSII(1) at the initial stage of HL exposure, allowing for sufficient binding of HliA/B to CP47m. This proposal is also supported by the results of 2D analyses of proteins from radioactively labelled and HL-treated WT and ∆Psb34 cells (Figs. 6 and 8). The mutant contains more labelled HliA/B in PSII(1) and especially in RC47 than WT, while the intensity of HliA/B labelling in CP47m is higher in WT (Fig. 6). Upon association of the CP47 module containing HliA/B with the D1/D2 RCII complex, Psb34 possibly replaces some HliA/B and allows their recycling and binding to CP47m for its sufficient photoprotection (for model see Fig. 11).

## Supplementary Information

Below is the link to the electronic supplementary material.

Supplementary file1 (PDF 1551 kb)
