# Supplementary material for: Psb34 protein modulates binding of high-light-inducible proteins to CP47-containing photosystem II assembly intermediates in the cyanobacterium Synechocystis sp. PCC 6803
Source: Photosynth Res. 2022 Mar 13;152(3):333–46. doi: 10.1007/s11120-022-00908-9 (PMC9458560; doi:10.1007/s11120-022-00908-9)
Supplement: Supplementary file 1 — Supplementary file1 (PDF 1551 kb) [file 11120_2022_908_MOESM1_ESM.pdf]

Supplementary data

The Psb34 protein modulates binding of high-light-inducible proteins to CP47-containing photosystem II assembly intermediates in the cyanobacterium *Synechocystis sp*. PCC 6803

Parisa Rahimzadeh Karvansara, Guillem Pascual-Aznar, Martina Bečková and Josef Komenda *

[1] Institute of Microbiology of the Czech Academy of Sciences, Centre Algatech, Laboratory of Photosynthesis, Opatovický mlýn, 37981 Třeboň, Czech Republic

*Correspondence: komenda@alga.cz; Tel.: (+420 384 240 431)

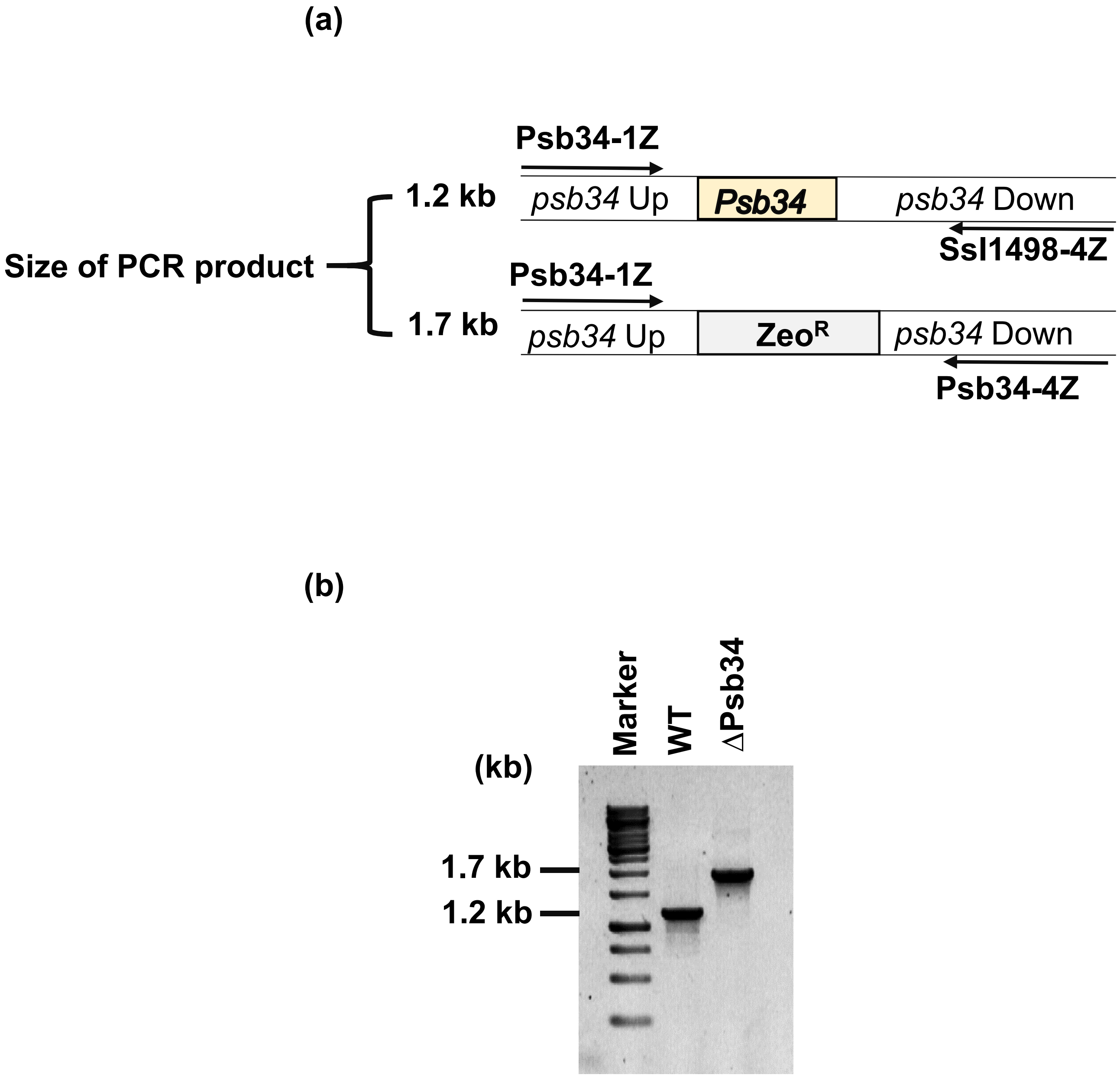

Fig. S1 Construction (a) and PCR verification (b) of the ΔPsb34 mutant strain. The strain was constructed by substituting the *psb34* (*ssl1498)* gene with the zeocin resistance cassette using the megaprimer method and segregation of the mutant was achieved by increasing the antibiotic concentration. The full segregation of the mutant was verified by PCR with the gene-specific primer pair Psb34-1Z (forward) and Psb34-1Z (reverse). For sequences of the primers see Supplementary Table S2.

```
                1       10        20        30        40        50
Tsl0063:        MRYTTDEGGRLNNFAIEPKVYQAQPWTPQQKVRAALLVGGGLLLVAGLVAIAVGVS
Ssl1498:      MNNYTKDDDGRLNNFAVEPKIYAADAPSKADKRNYLIMAAITVVLVAGLIAVAVVASGAST
HliA:        MTTRGFRLDQDNRLNNFAIEPEVYVDSSVQAGWTKYAEKMNGRFAMIGFASLLIMEVVTGHGVIGWLNSL
HliB:        MTSRGFRLDQDNRLNNFAIEPPVYVDSSVQAGWTEYAEKMNGRFAMIGFVSLLAMEVITGHGIVGWLLSL
```

Fig. S2 Comparison of amino acid sequences of the Tsl0063 protein from *Thermosynechococcus elongatus* found in PSII assembly intermediates (PDB codes 7NHP and 7DXH) and Ssl1498, HliA (Ssl2542) and HliB (Ssr2595) from *Synechocystis sp.* PCC 6803. The N-terminal sequences homologous among proteins are underlined. The transmembrane helix of proteins (in red) was determined for Tsl0063 and predicted accordingly for other three proteins. The Chl-binding motif of Hlips missing in Tsl0063 and Ssl1498 is double underlined. The amino acid residues of Tsl0063 forming hydrogen bonds with D1 (E7, yellow), CP47 (L11, A15, E17, green), PsbL (N13, grey) and PsbH (Y21, A23, cyan) are shown and with exception of E7 and A23 are shared with HliA/B.

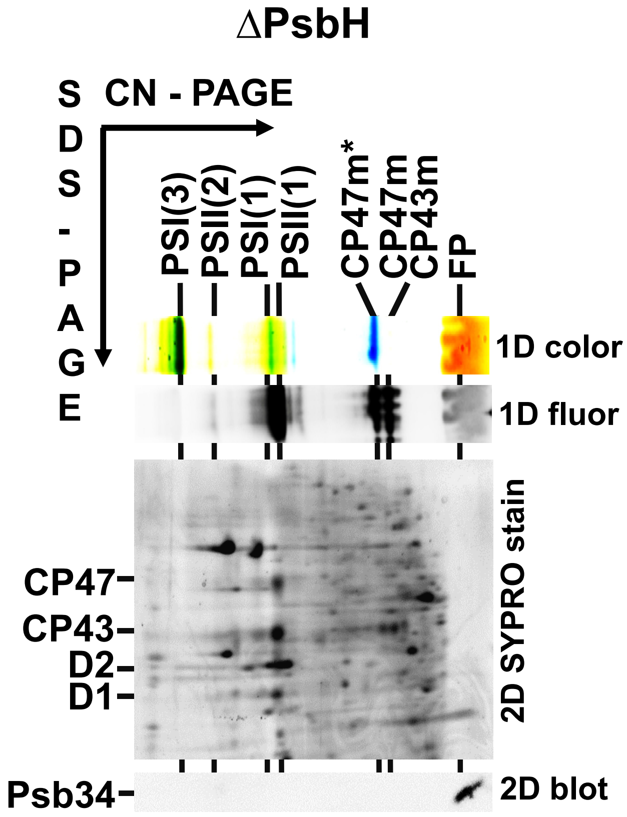

Fig. S3 2D analysis of membrane proteins of the ΔPsbH strain. Membranes isolated from the strain were analysed using 2D-CN/SDS-PAGE. After the first dimension, the gel was photographed (1D color) and scanned for Chl fluorescence (1D fluor). After separation in the second dimension, the 2D gel was stained with SYPRO Orange (2D SYPRO stain), blotted onto a PVDF membrane (2D blot) and Psb34 was detected with the specific antibody. Designation of complexes: PSI(3) and PSI(1), trimeric and monomeric Photosystem I; PSII(2) and PSII(1), dimeric and monomeric PSII core complexes; CP47m*, CP47 module containing Psb35; CP47m and CP43m, unassembled CP47 and CP43 modules; FP, free pigments. The loaded sample contained 5 µg of Chl.

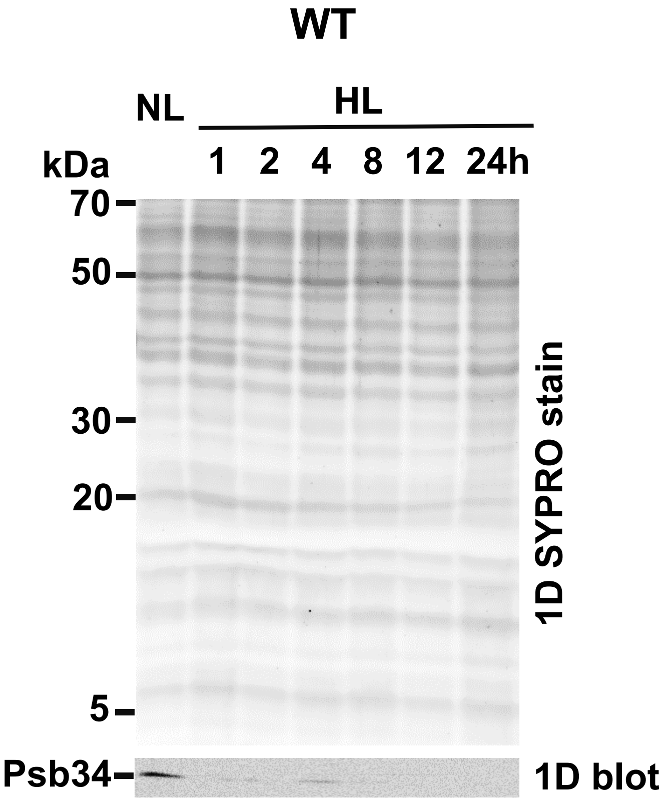

Fig. S4 1D analysis of membrane proteins from WT exposed to NL (40 µmol photons $m^{-2}s^{-1}$) and HL (500 µmol photons $m^{-2}s^{-1}$) for 1, 2, 4, 8, 12, and 24 hours. Membranes isolated from the strains were analysed using SDS PAGE and 1D gel was stained with SYPRO Orange (1D SYPRO stain), blotted onto a PVDF membrane (1D blot) and Psb34 was detected with the specific antibody. SYPRO-stained gel documents the equal loading of the samples. Each loaded sample contained 5 µg of Chl.

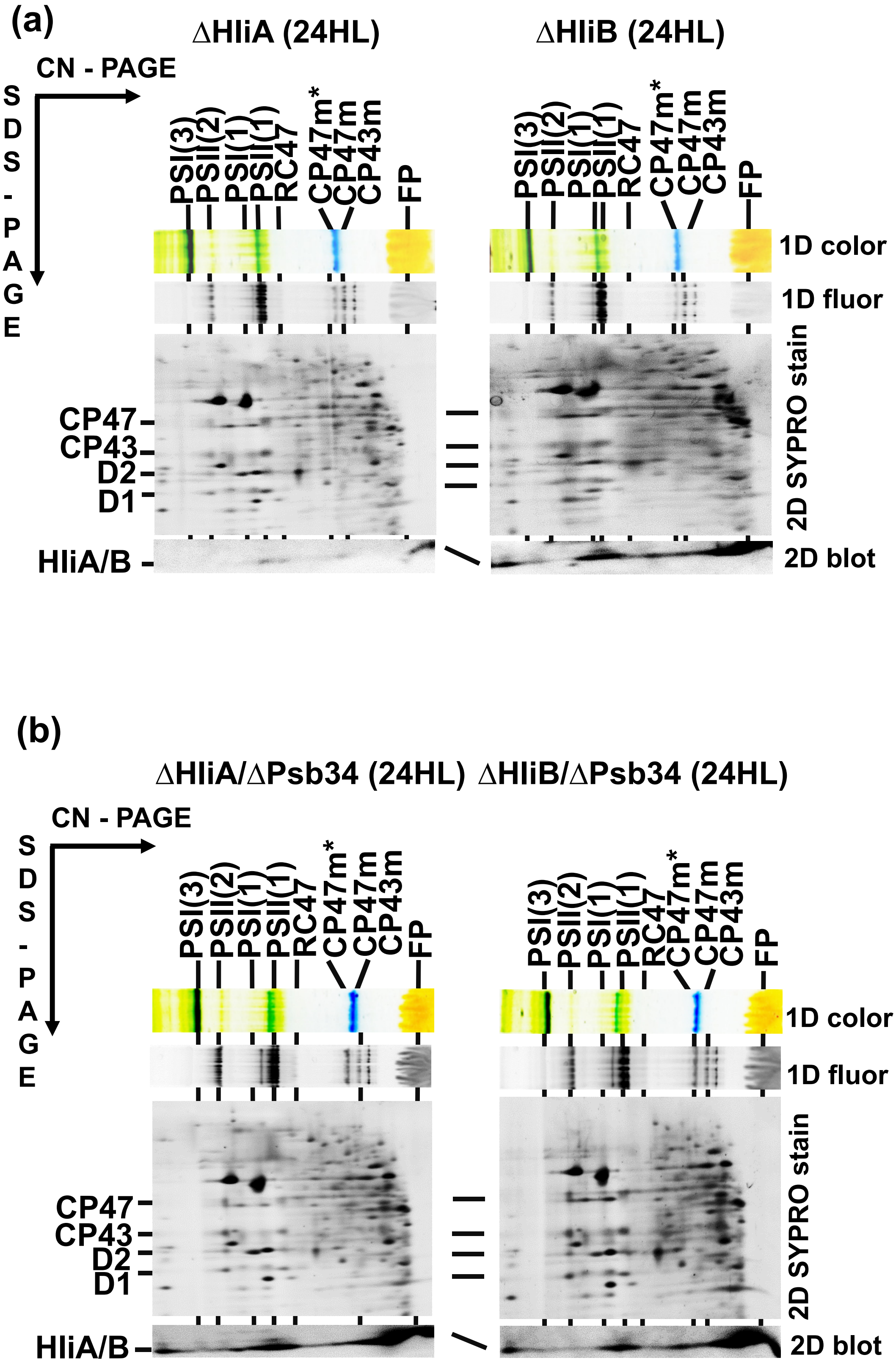

Fig. S5 2D analysis of membrane proteins from ΔHliA and ΔHliB cells (a) and ΔHliA/ΔPsb34 and ΔHliB/ΔPsb34 cells exposed to HL for 24 hours (24HL). Membranes isolated from the strains were analysed using 2D-CN/SDS-PAGE. After the first dimension, the gels were photographed (1D color) and scanned for Chl fluorescence (1D fluor). After separation in the second dimension, the 2D gels were stained with SYPRO Orange (2D SYPRO stain), blotted onto a PVDF membrane (2D blot) and HliA/B were detected with the specific antibodies. Designation of complexes as in Fig. S3. Each loaded sample contained 5 μg of Chl.

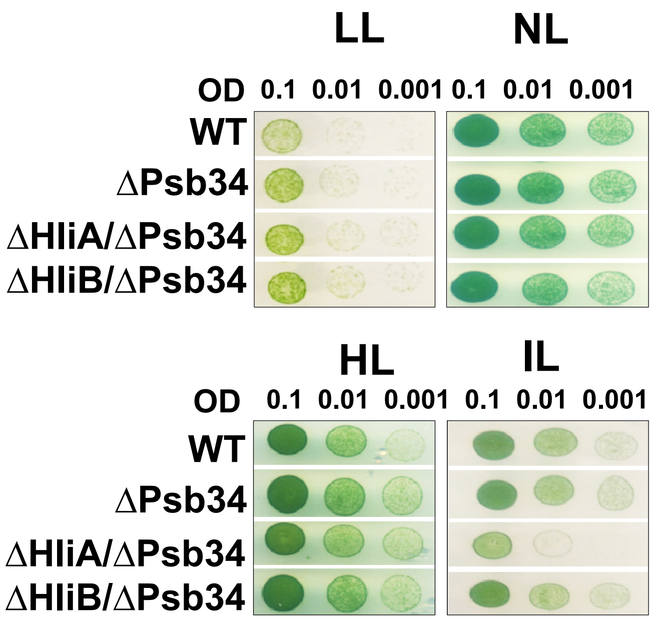

Fig. S6 Growth of wild type (WT), ΔPsb34, ΔHliA/ΔPsb34 and ΔHliB/ΔPsb34 mutants under various light conditions. Cells were spotted on agar plates containing BG-11 and 10 mM TES/NaOH, pH 8.0 and exposed to LL, NL, HL and IL for 8 days as described in Materials and methods.

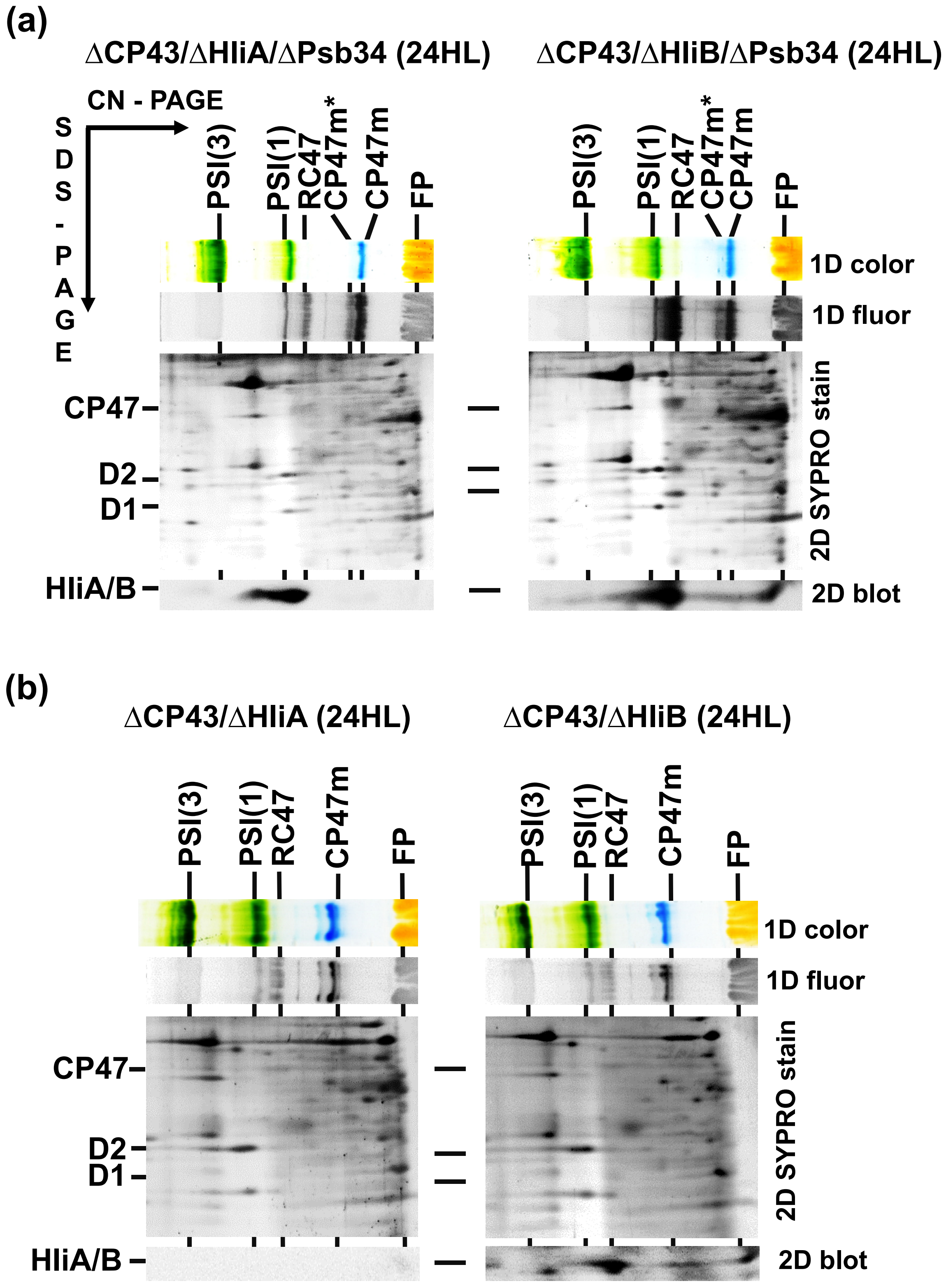

Fig. S7 2D analysis of membrane proteins from ΔCP43/ΔHliA/ΔPsb34 and ΔCP43/ΔHliB/ΔPsb34 (a) and ΔCP43/ΔHliA and ΔCP43/ΔHliB (b) cells exposed to HL for 24 hours (24HL). Membranes isolated from the strains were analysed using 2D-CN/SDS-PAGE. After the first dimension, the gels were photographed (1D colour) and scanned for Chl fluorescence (1D fluor). After separation in the second dimension, the 2D gels were stained with SYPRO Orange (2D SYPRO stain) and blotted onto a PVDF membrane (2D blot), and HliA/B were detected with the specific antibody. Designation of complexes as in Fig. S3. Each loaded sample contained 3.5 μg of Chl.

Table S1: ***Synechocystis*** **strains used in the study.**

Strain	Deleted/modified gene	Reference
WT	*Synechocystis GT-P*	(Tichý, Bečková et al. 2016)
ΔPsb34	*Δssl1498*	This study
ΔPsbH	*Δssl2598*	(Mayes et al. 1993)
ΔCP47	*Δsll0906*	(Eaton-Rye and Vermaas 1991)
ΔCP43	*Δsll0849*	(Vermaas et al. 1988)
ΔHliA	*Δssl2542*	(Konert et al. 2021)
ΔHliB	*Δssr2595*	(Xu et al. 2004)
ΔCP43/ΔPsb34	*Δsll0849/Δssl1498*	This study
ΔCP43/ΔHliA	*Δsll0849/Δssl2542*	This study
ΔCP43/ΔHliB	*Δsll0849/ Δssr2595*	This study
ΔHliA/ΔPsb34	*Δssl2542/Δssl1498*	This study
ΔHliB/ΔPsb34	*Δssr2595/Δssl1498*	This study
ΔCP43/ΔHliA/ΔPsb34	*Δsll0849/Δssl2542/Δssl1498*	This study
ΔCP43/ΔHliB/ΔPsb34	*Δsll0849/Δssr2595/Δssl1498*	This study
Psb28-FLAG/ΔPsb28	*sll1398-FLAG/Δsll1398*	(Bečková et al. 2017)
FLAG-Psb34/ΔPsb34	*FLAG-ssl1498/Δssl1498*	This study

Table S2: List of primers used in this study.

Strain	Primer name	Primer sequence
**ΔPsb34**	Psb34-1Z	tagcccttggggtaggattc
	Psb34-2Z	acattaattgcgttgcgctcactgcGCCGTCCATCATCGTCTTTA*
	Psb34-3Z	caacttaatcgccttgcagcacatCGGGGCTAGCACCTAAtttt*
	Psb34-4Z	tcgggaaagaattctgcatc
**FLAG-Psb34**	Not_1498_N_F	ctagaggcggccgcaaacaactatactaaagac
	BamH_1498_N_R	gaggacggatccttaggtgctagccccggaagc

*Capital letters represent the sequence to amplify zeocin cassette.

Supplementary list of references:

Bečková M, Gardian Z, Yu J et al (2017) Association of Psb28 and Psb27 proteins with PSII-PSI supercomplexes upon exposure of Synechocystis sp. PCC 6803 to high light. Mol Plant 10:62-72. https://doi.org/10.1016/j.molp.2016.08.001

Eaton-Rye JJ, Vermaas WF (1991) Oligonucleotide-directed mutagenesis of psbB, the gene encoding CP47, employing a deletion mutant strain of the cyanobacterium Synechocystis sp. PCC 6803. Plant Mol Biol 17:1165–1177.. https://doi.org/10.1007/BF00028733

Konert M, Wysocka A, Koník P et al. (2021) High-light-inducible proteins HliA and HliB: pigment binding and protein-protein interactions. Photosynth Res, doi: 10.1007/s11120-022-00904-z

Mayes SR, Dubbs JM, Vass I (1993) Further characterization of the psbH locus of Synechocystis sp. PCC 6803: inactivation of psbH impairs Q_A to Q_B electron-transport in photosystem 2. Biochem 32:1454–1465. https://doi.org/10.1021/bi00057a008

Tichý M, Bečková M, Kopečná J et al (2016) Strain of Synechocystis PCC 6803 with aberrant assembly of photosystem II contains tandem duplication of a large chromosomal region. Front Plant Sci 7:648. https://doi.org/10.3389/fpls.2016.00648

Vermaas WFJ, Ikeuchi M, Inoue Y (1988) Protein composition of the photosystem II core complex in genetically engineered mutants of the cyanobacterium Synechocystis sp. PCC 6803. Photosynth Res 17:97–113. https://doi.org/10.1007/978-94-009-2269-3_18

Xu H, Vavilin D, Funk C et al (2004). Multiple deletions of small Cab-like proteins in the cyanobacterium Synechocystis sp. PCC 6803: Consequences for pigment biosynthesis and accumulation. J Biol Chem 279:27971–27979. https://doi.org/10.1074/jbc.M403307200
